# Effects of Mild and Severe Knee Joint Pain on Various Activities of Daily Living in the Female Elderly

**DOI:** 10.1155/2013/989508

**Published:** 2013-10-02

**Authors:** Hiroki Sugiura, Shinichi Demura

**Affiliations:** Graduate School of Natural Science and Technology, Kanazawa University, Kakuma, Kanazawa, Ishikawa 920-1192, Japan

## Abstract

This study aimed to examine the differences in the ability to perform various activities of daily living (ADLs) among groups with various knee problems. The participants consisted of 328 elderly females (age 60–94; mean age 76.1 years; standard deviation 6.2). The subjects were classified into three groups: those without knee pain, those with mild knee pain, and those with severe knee pain. ADLs with markedly higher (>97%) and lower (<38%) achievement rates in the group without knee pain were not significantly different among the three groups. Achievement rates of 40%–97% for ADLs were significantly lower in the group with severe knee pain than in the group without knee pain. In addition, the groups with mild and severe knee pain demonstrated significantly lower achievement rates of ascending and descending stairs and sitting up than the group without knee pain. In conclusion, regardless of the presence of absence of mild or severe knee pain, some ADLs are difficult to achieve, while others are easy. The elderly with severe knee pain find it difficult to achieve many ADLs. In addition, it is difficult for the elderly with mild and severe knee pain to ascend and descend stairs and to sit up.

## 1. Introduction

In old age, physical functions such as leg strength, balance, and mobility of the leg joints decrease markedly with age. Prevention of a reduction in these physical functions is crucial to allow the elderly to continue a healthy and independent daily life [1, 2]. Demura and Sato [3] reported that the ability to live independently should be assessed in the elderly. In addition, Sato et al. [4] reported that the ability to achieve activities of daily living (ADLs), rather than the ability to perform physical functions at maximum exertion, should be assessed for the elderly. In brief, it is important for the elderly to retain the ability to achieve ADLs at above a certain level to maintain an independent daily life [4, 5].

Among different leg joints, knee joints have the greatest load-bearing capacity, and double the usual load of body weight is imposed on each knee joint when standing on one leg or when walking [6]. Knee joints are important for achieving independence in ADLs [7]. Recently, the number of elderly who suffer from mild or severe knee pain has increased [8]. O'Reilly et al. [9] and McAlindon et al. [10] have reported that approximately 25% of the elderly have mild or severe knee pain. In addition, Peat et al. [8] reported that 50% of the elderly with knee osteoarthritis, which is the main cause of mild and severe knee pain, do not feel subjective pain. However, they are more likely to suffer mild or severe knee pain in the near future. Peat et al. [8] and Oida and Nakamura [11] reported that the prevalence of mild and severe knee pain is high in the female elderly. It is inferred that the number of elderly females with mild or severe knee pain will increase.

The ADL survey was used to assess the physical ability which the elderly are necessary to pass independent daily life. Sugiura and Demura [12] reported that the elderly subjects with knee pain had inferior locomotion movements and posture change than those without knee pain. However, each ADL has respective different difficulty [13]. In short, the difficulty level exists from low activities to high ones. We have assumed that activities of a high difficulty level for the elderly without knee pain would also be of high difficulty for the elderly with mild or severe knee pain. However, the latter group of elderly may even find activities with a low difficulty level more challenging than the former group of elderly. In addition, we have assumed that the elderly with severe knee pain would find it more difficult to perform various ADLs than the elderly with mild knee pain, who are considered to be an auxiliary group of the elderly with severe knee pain. This study, therefore, aimed to examine the differences in the ability to achieve various ADLs among the following groups of female elderly: those without knee pain, those with mild knee pain, and those with severe knee pain.

## 2. Method

### 2.1. Participants

Even if the elderly have orthopedic abnormalities, approximately 50% do not feel subjective knee pain [8, 14]. Peat et al. [8] reported that it is necessary to focus on pain in the knee joints because it is common among the elderly and varies in type and cause. In this study, elderly individuals who selected Yes in response to the question “Do you have an articular disorder (ankle, knee, hip joint)? (choice: Yes and No)” and Right, Left, or Both in response to the question “Do you have knee pain or disorders? (choice: Right, Left, Both, and No)” were defined as patients with knee pain. Individuals who selected No in response to both questions were defined as those without knee pain. Mild and severe knee pain were judged using the Japanese edition knee function scale [15] on the basis of the Western Ontario and McMaster Universities Osteoarthritis Index [16]. According to this assessment, individuals with over 210 points and those with less than 210 points were judged to have severe knee pain and mild knee pain, respectively, [11].

Participants consisted of 328 female elderly people (age 60–94; mean age 76.1 years; standard deviation 6.2). The subjects were classified into the following five groups: 168 persons without knee pain (no knee pain (G1) group), 116 persons with mild knee pain (mild unilateral knee pain (G2) group *n* = 75 and mild bilateral knee pain (G3) group *n* = 41), and 44 persons with severe knee pain (severe unilateral knee pain (G4) group *n* = 21 and severe bilateral knee pain (G5) group *n* = 23). Forty subjects in the G2 group and 11 subjects in the G4 group had right knee pain. In addition, subjects participated in health classes or social educational activities hosted by municipal governments and engaged in social activities at least once per week or on alternate weeks. In brief, these individuals could achieve ADLs independently.

Table 1 shows the basic statistics (age, height, and body weight) of subjects in each group. The purpose and procedures of this study were explained in detail to all participants before their informed consent was obtained. The present experimental protocol was approved by the Ethics Committee on Human Experimentation of Faculty of Human Science, Kanazawa University (Ref. number 2012-12).

### 2.2. ADL

The ADL survey was prepared to confirm whether the elderly can safely participate in a physical fitness test administered by the Ministry of Education, Culture, Sports, Science and Technology of Japan (Table 2). This survey consists of four domains: locomotion (walking, running, jumping across a ditch, ascending and descending stairs, and convey), posture change (sitting up and standing up from the floor), stability (standing on one foot with eyes open, standing in a bus or a train, and dressing while standing), and manipulation (buttoning a shirt and placing a Japanese mattress into and removing it out of a closet), and the degree of achievement of ADLs required for independent life was evaluated according to these 12 items [4, 5]. Each item consisted of three different difficulty levels, with subjects selecting the appropriate level for each ADL item. For example, subjects could select from among the following categories “1: approximately 5–10 min,” “2: approximately 20–40 min,” and “3: over an hour” for question 1 (How long can you walk without taking rest?), it was judged that when a subject selected “3,” the individual could also achieve the task at levels “1” and “2”, when a subject selected “2,” the individual could also achieve the task at a level of “1”, and when a subject selected “1,” the individual could not achieve the task at a level of “2” and “3.” Twenty-seven movements among 36 movements, excluding categories such as “cannot run” and “cannot jump” were used for analysis (Table 3). In this study, movements with a higher achievement rate were judged to be those involving lower difficulty.

### 2.3. Statistical Analysis

Mean differences of age, height, and body weight were examined by ANOVA. The one-dimensional nature of ADLs measured in the G1 group was examined by a reproducibility coefficient and a scaling coefficient of Guttman [13, 17], which was based on the order of the achievement rates. The achievement rates of ADL were examined by a difference of proportion test. Scheffe's test was used for multiple comparisons when significant differences were found. Relationships between the presence or absence of mild or severe knee pain and achievement rates of ADLs were examined on the basis of the association coefficient of Cramer. The significance level in this study was set at *P* < 0.05, which was adjusted by Scheffe's method.

## 3. Results

Table 1 shows the basic statistics of age, height, and body weight in the groups with no knee pain (G1), mild unilateral knee pain (G2), mild bilateral knee pain (G3), severe unilateral knee pain (G4), severe bilateral knee pain (G5), and the test results and their mean values. The results of one-way ANOVA showed a significant difference only in the body weight. Participants from the severe knee pain group (G4 + G5) were significantly heavier than those in the G1 group. However, no significant difference was observed in the body weight between the participants in the G2 and G3 groups and those in the G4 and G5 groups.

**Figure 1:**
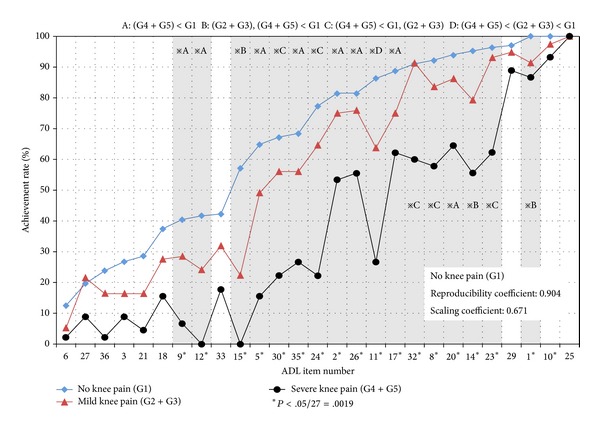
The one-dimensional nature of ADLs measured in the G1 group and difference in achievement rates of ADLs among group.

Figure 1 shows the order of achievement rates of various ADLs in the G1, G2 + G3, and G4 + G5 groups. The reproducibility coefficient of the G1 group was very high (0.904), and the scaling coefficient was 0.671.

Table 3 shows the basic statistics of achievement rates of ADLs in G1, G2, G3, G4, and G5 groups. A significant difference was found among groups in 18 of 27 movements. Results of multiple comparison tests showed that differences between the G2 and G3 groups as well as between the G4 and G5 groups were insignificant for all movements. Hence, both the G2 and G3 groups and the G4 and G5 groups were pooled, and linear comparisons were made between the resulting three groups (no knee pain G1; mild knee pain G2 + G3; and severe knee pain G4 + G5). ADL items 1, 3, 6, 10, 18, 21, 25, 27, 29, and 36 for which the achievement rates were <37.5% and >97% in the G1 group showed no significant differences among the groups. ADL items 2, 5, 8, 9, 11, 12, 14, 15, 17, 20, 23, 24, 26, 30, 32, 33, and 35, with achievement rates between 40% and 97% in the G1 group, showed significant differences. The achievement rate for item “11. Ascend stairs without using the banister (slowly)” was significantly lower in the G4 + G5 group, followed by the G2 + G3 and G1 groups. The achievement rates of movements 2, 5, 9, 12, 17, 20, 26, and 35 were lower in the G4 + G5 group than in the G1 group; those of movements 1, 14, and 15 were lower in the G4 + G5 and G2 + G3 groups than in the G1 group; and those of movements 8, 23, 24, 30, and 32 were lower in the G4 + G5 group than in the G1 and G2 + G3 groups. The achievement rate of “33. Can carry baggage of approximately 10 kg for 10 m” was not significantly different among the three groups. Association between the presence or absence of mild or severe knee pain and the achievement rate of ADLs was moderate (*V*: 0.30–0.45) in the 12 movements of items 1, 5, 8, 11, 12, 14, 15, 20, 23, 24, 30, and 32, but it was low (*V*: 0.11–0.29) in the other movements.

## 4. Discussion

Values for body weight were higher in the group with severe knee pain than in the group with no knee pain. This result was similar to that in previous studies [18]. In elderly individuals with severe knee pain, body weight increased because of the limited physical activity due to the knee pain. Training to increase strength around the knee joints is useful in such cases, and exercise that places no excessive burden on the knee joints is useful as a precaution to prevent the onset or exacerbation of knee pain [19]. This is particularly desirable for elderly individuals with severe knee pain to perform the aforementioned training or exercise.

Achievement rates of ADLs in the elderly without knee pain (the general elderly) were examined using the one-dimensional nature of Guttman in this study. This allows a graded evaluation of movements because the difficulty level for such elderly is different. Among 27 movements, the difficulty level of the following five movements was high (12%–29%): “10 min running,” “elaborate movement using one hand,” “trunk posture change several times,” “endurance walking,” and “standing within a bus or train without using the handhold, except when departing and stopping,” and the difficulty level was very low (91%–100%) in the following nine movements: “elaborate movement using both hands,” “short time walking,” “put off a light weight,” “slow ascent and descent of stair,” “dressing while standing,” “standing within a bus or train using the handhold,” “sitting up using the hands,” “jumping across a ditch of 30 cm,” and “transportation of a weight of 5 kg” (Figure 1 and Table 3). The reproducibility coefficient of Guttman for the achievement rates of the 27 movements was very high (0.904). When this value exceeds 0.9, the approximation with the complete measure is generally guaranteed [13, 20]. This suggests that the ability of the general elderly to achieve ADLs can be estimated as a one-dimensional continuum by 27 movements.

It was assumed that the elderly with mild or severe bilateral knee pain find it more difficult to achieve ADLs than those with mild or severe unilateral knee pain and that the achievement rates would be reduced in elderly with mild knee pain in bilateral knee and severe knee pain affecting bilateral knee. However, no significant difference was found between the G2 and G3 groups and between the G4 and G5 groups for all ADLs. The subjects were those elderly who can achieve ADLs independently. Many ADLs, such as walking, ascending and descending stairs, and standing up, involve both legs equally. They may have achieved ADLs even while enduring pain. From our results, we can deduce that the elderly with mild or severe knee pain affecting bilateral knee are similar to the elderly with mild or severe knee pain affecting in unilateral knee in terms of achieving some ADLs or the difficulty they experience in achieving these activities. Hence, the groups with mild unilateral knee pain (G2) and with mild bilateral knee pain (G3) (the mild knee pain group G2 + G3) and the groups with severe unilateral knee pain (G4) and severe bilateral knee pain (G5) (the severe knee pain group G4 + G5) were pooled and analyzed as such in this study.

The difficulty level of six movements (items 6, 27, 36, 3, 21, and 18; see Table 3) was higher in all groups (G1: 12%–38%, G2 + G3: 5%–28%, and G4 + G5: 2%–16%). The achievement rate for these movements was under 40% in G1 group. However, the difference among the groups was insignificant. Strength of the legs [21–23] and body trunk [24, 25], muscle endurance [26], ability to balance [27, 28], and cardiorespiratory function [29, 30] decrease markedly with age. A decrease in physical functions with age greatly affects the difficulty level for various ADLs. Our study indicates that the difficulty of the abovementioned ADLs is considered to be high for the elderly, regardless of whether they have mild or severe knee pain.

The difficulty level of four movements (items 9, 12, 33, and 15; see Table 3) was higher in the G4 + G5 group (0%–16%), but moderate in the other two groups (G1: 40%–65% and G2 + G3: 22%–50%). The achievement rate for these movements ranged between 40% and 65% in the G1 group. Morrison [6] reported that during standing on one leg or walking, double the usual load of body weight is imposed on the knee joints. Knee joints are, therefore, important for efficient achievement of ADLs. The elderly with severe knee pain may be limited in achievement of those ADLs that impose a heavy burden on the knee joints, because of severe knee pain, and they find it difficult to achieve these activities. In addition, the difficulty level experienced by the G2 + G3 and G4 + G5 groups were increased for movements involved in “Stand up from a Japanese style sitting posture without using hands” compared with that experienced by the G1 group (G1 group 57.1%; G2 + G3 group 22.4%; and G4 + G5 group 0%). Because rising up from a Japanese style sitting position (Seiza) requires greater mobility of the knee joints than for rising up from a chair, individuals with mild knee pain may find it difficult to achieve, similar to individuals with severe knee pain due to the large burden placed on the knee joints by this movement. On the other hand, it was considered that the movement involved in “Carry baggage of approximately 10 kg for 10 m” would also be achieved with different levels of success among the groups because of imposing a heavy burden on the knee joints by carrying a load. However, the difference in this case was insignificant (G1 group 42.3%; G2 + G3 group 31.9%; and G3 + G4 group 17.8%). Sugiura and Demura [31] reported that the elderly with mild knee pain may be able to walk while enduring pain for a short distance. This infers that although this movement is experienced as quite difficult by individuals with severe knee pain, many elderly in general feel that this is difficult to achieve.

The difficulty level of three movements (items 30, 35, and 24; see Table 3) was relatively lower in the G1 (67%–78%) and G2 + G3 group (22%–50%). The achievement rate for these movements ranged between 67% and 78% in the G1 group. The majority of both groups were considered to be able to achieve these movements. However, these movements appeared to be more difficult for the G3 + G4 group (22.2%–26.7%). On the other hand, severe knee pain may affect not only locomotion but also lead to a reduction in the frequency of use of the upper limbs and trunk. Thus, even if the difficulty level of a particular ADL was low for the general elderly or for the elderly with mild knee pain, the difficulty level may be high for the elderly with severe knee pain.

The majority of individuals (53–97%) in any group could achieve the eight movements (items 2, 26, 17, 32, 8, 20, 14, and 23; see Table 3), but the difficulty level was higher in the G4 + G5 group (53%–65%) than in the G1 group (81%–97%). The achievement rate for these movements ranged between 81% and 97% in the G1 group. The elderly with severe knee pain may find it difficult to achieve the movements that impose a heavy burden on the knee joints. In addition, we found that the difficulty level of “Ascend stairs without using the banister, slowly” was significantly higher in the G4 + G5 group, followed by the G2 + G3 group and the G1 group (G1 group 86.3%; G2 + G3 group 63.8%; and G4 + G5 group 26.7%). Maruyama [32] reported that ascending and descending the stairs imposes a larger burden on the knee joints because the knee joints are used as a fulcrum point, inferring that such a movement is difficult to achieve for the elderly with severe knee pain. The elderly with mild and severe knee pain need to reduce the burden of weight imposed on the knee joints when ascending and descending the stairs during daily life.

The difficulty level of three movements (items 1, 10, and 25; see Table 3) was lower in all groups (G1: 100%, G2 + G3: 91%–100%, and G4 + G5: 86%–100%). The achievement rate for these movements 100% in G1 group. Thus, most elderly people could achieve them (87%–91%). This indicates that the elderly people in the present study could achieve ADLs independently in spite of having mild or severe knee pain (see subjects in Section 2).

## 5. Conclusion

ADLs with which the female elderly in general find great or little difficulty are also the activities that the elderly with mild and severe knee pain find to be particularly difficult or easy. Thus, regardless of the presence or absence of mild or severe knee pain, some ADLs are difficult to achieve, while others are easy. The elderly with severe knee pain find it difficult to achieve many ADLs. In particular, it is challenging for the elderly with mild and severe knee pain to ascend and descend stairs and to rise up from a sitting position.

## Figures and Tables

**Table 1:** The basic statistics for age, height, and body weight of subjects in each group.

	No knee pain (G1: *n* = 168)				Mild unilateral knee pain (G2: *n* = 75)				Mild bilateral knee pain (G3: *n* = 41)				Severe unilateral knee pain (G4: *n* = 21)				Severe bilateral knee pain (G5: *n* = 23)				ANOVA		Scheff's post hoc
	M	SD	MAX	MIN	M	SD	MAX	MIN	M	SD	MAX	MIN	M	SD	MAX	MIN	M	SD	MAX	MIN	*F *	*P *	G1, (G2 + G3), (G4 + G5)
Age (yr)	75.4	6.7	88	62	76.8	6.0	94	60	76.4	5.2	86	60	76.7	5.1	87	68	77.5	5.7	85	60	1.10	0.36	—
Height (cm)	147.8	6.2	164.5	132.5	147.2	6.2	161.4	132.5	147.7	5.7	160.0	138.4	149.1	5.0	156.0	138.0	146.7	5.9	159.4	140.0	0.47	0.76	—
Weight (kg)	48.40	7.6	69.6	32.1	49.32	12.4	70.9	32.5	53.76	7.4	69.1	40.8	54.69	6.9	71.0	39.0	53.42	6.6	68.8	41.7	3.08*	0.02	G1 < (G4 + G5)

Note: *P < 0.05.

**Table 2:** ADL test.

ADL domain	ADL items	ADL answered number	Movements
Locomotion	(1) How long can you walk without taking a rest?	(1) About 5–10 minutes	(1) Walk for approximately 5–10 min without taking rest
		(2) About 20–40 minutes	(2) Walk for approximately 20–40 min without taking rest
		(3) Over an hour	(3) Walk for over an hour without taking rest
Locomotion	(2) How long can you run without taking a rest?	(1) Impossible	(4) Cannot run without taking rest
		(2) About 3–5 minutes	(5) Run for approximately 3–5 min without taking rest
		(3) Over 10 minutes	(6) Run for >10 min without taking rest
Locomotion	(3) How wide a gutter can you jump over?	(1) Impossible	(7) Cannot jump across a ditch
		(2) About 30 cm	(8) Jump across a ditch of approximately 30 cm
		(3) About 50 cm	(9) Jump across a ditch of approximately 50 cm
Locomotion	(4) How do you go up the stairs?	(1) Need banister	(10) Ascend stairs using the banister
		(2) Slowly (no use banister)	(11) Ascend stairs without using the banister (slowly)
		(3) Fleetly (no use banister)	(12) Ascend stairs without using the banister (fleetly)
Posture change	(5) How do you stand up from a Japanese style sitting posture, that is, Seiza?	(1) Impossible	(13) Cannot stand up from a Japanese style sitting posture
		(2) Use hands	(14) Stand up from a Japanese style sitting posture using hands
		(3) Stand without use of hands	(15) Stand up from a Japanese style sitting posture without using hands
Stability	(6) How many seconds can you stand on one leg with eyes open?	(1) Impossible	(16) Cannot stand on one leg with eyes open
		(2) About 10–20 seconds	(17) Stand on one leg with eyes open for approximately 10–20 seconds
		(3) Over 30 seconds	(18) Stand on one leg with eyes open for >30 s
Stability	(7) Can you remain standing on a bus or train?	(1) Impossible	(19) Cannot stand within a bus or train
		(2) Using the handhold	(20) Standing within a bus or train using the handhold
		(3) Without using the handhold (except departing and stopping)	(21) Standing within a bus or train without using the handhold (except departing and stopping)
Stability	(8) Can you put on trousers or a skirt while standing?	(1) Sitting only	(22) Cannot put on trousers or a skirt while standing
		(2) Use the handhold	(23) Put on trousers or a skirt while standing using a handhold
		(3) Without use the handhold	(24) Put on trousers or a skirt while standing without using a handhold
Manipulation	(9) Can you do or undo the front buttons of a shirt?	(1) Both hands (slowly)	(25) Do or undo the front buttons of a shirt using both hands (slowly)
		(2) Both hands (freely)	(26) Do or undo the front buttons of a shirt using both hands (fleetly)
		(3) One hand	(27) Do or undo the front buttons of a shirt using one hand
Manipulation	(10) Can you roll up and put away the bedding?	(1) Impossible	(28) Cannot roll up and put away the bedding
		(2) Lightweight only	(29) Roll up and put away lightweight bedding
		(3) Heavy weight	(30) Roll up and put away heavy weight bedding
Locomotion	(11) What weight of baggage can you carry 10 m?	(1) Impossible	(31) Cannot carry baggage
		(2) About 5 kg	(32) Can carry baggage of approximately 5 kg for 10 m
		(3) About 10 kg	(33) Can carry baggage of approximately 10 kg for 10 m
Posture change	(12) Can you raise your upper body without using your hands when lying down and facing up?	(1) Impossible	(34) Cannot raise upper body
		(2) About 1-2 times	(35) Raise the upper body approximately 1-2 times without using hands when lying down and facing up
		(3) About 3-4 times	(36) Raise the upper body approximately 3-4 times without using hands when lying down and facing up

**Table 3:** Difference in achievement rates of ADLs among groups.

ADL items	No knee pain	Mild unilateral knee pain	Mild Bilateral knee pain	Severe Unilateral knee pain	Severe Bilateral knee pain	*χ* ^2^	*V *	Mild knee pain	Severe knee pain	Scheff's post hoc		
	(G1: *n* = 168)	(G2: *n* = 75)	(G3: *n* = 41)	(G4: *n* = 21)	(G5: *n* = 23)			G2 + G3	G4 + G5	G2, G3	G4, G5	G1, (G2 + G3), (G4 + G5)
(6) Run for >10 min without taking rest	12.5%	6.7%	2.4%	4.5%	0.0%	8.29	0.16	5.2%	2.2%	—	—	—
(27) Do or undo the front buttons of a shirt using one hand	19.6%	22.7%	19.5%	9.1%	8.7%	3.72	0.11	21.6%	8.9%	—	—	—
(36) Raise the upper body approximately 3-4 times without using hands when lying down and facing up	23.8%	10.7%	26.8%	0%	4.3%	16.29	0.22	16.4%	2.2%	—	—	—
(3) Walk for over an hour without taking rest	26.8%	21.3%	7.3%	13.6%	4.3%	12.71	0.20	16.4%	8.9%	—	—	—
(21) Standing within a bus or train without using the handhold (except departing and stopping)	28.6%	17.3%	14.6%	9.1%	0%	15.42	0.22	16.4%	4.4%	—	—	—
(18) Stand on one leg with eyes open for >30 s	37.5%	25.3%	31.7%	13.6%	17.4%	9.55	0.17	27.6%	15.6%	—	—	—
(9) Jump across a ditch of approximately 50 cm	40.5%	26.7%	31.7%	13.6%	0.0%	20.88*	0.25	28.4%	6.7%	—	—	(G4 + G5) < G1
(12) Ascend stairs without using the banister (fleetly)	41.7%	25.3%	22.0%	0%	0%	32.34*	0.31	24.1%	0%	—	—	(G4 + G5) < G1
(33) Can carry baggage of approximately 10 kg for 10 m	42.3%	28.0%	39.0%	22.7%	13.0%	12.08	0.19	31.9%	17.8%	—	—	—
(15) Stand up from a Japanese style sitting posture without using hands	57.1%	24.0%	19.5%	0%	0%	66.43*	0.45	22.4%	0%	—	—	(G2 + G3), (G4 + G5) < G1
(5) Run for approximately 3–5 min without taking rest	64.9%	57.3%	34.1%	22.7%	8.7%	42.09*	0.36	49.1%	15.6%	—	—	(G4 + G5) < G1
(30) Roll up and put away heavy weight bedding	67.3%	54.7%	58.5%	31.8%	13.0%	31.27*	0.31	56.0%	22.2%	—	—	(G4 + G5) <G1, (G2 + G3)
(35) Raise the upper body approximately 1-2 times without using hands when lying down and facing up	68.5%	56.0%	56.1%	36.4%	17.4%	27.57*	0.29	56.0%	26.7%	—	—	(G4 + G5) < G1
(24) Put on trousers or a skirt while standing without using a handhold	77.4%	65.3%	63.4%	27.3%	17.4%	48.26*	0.39	64.7%	22.2%	—	—	(G4 + G5) <G1, (G2 + G3)
(2) Walk for approximately 20–40 min without taking rest	81.5%	78.7%	68.3%	59.1%	47.8%	17.54*	0.23	75.0%	53.3%	—	—	(G4 + G5) < G1
(26) Do or undo the front buttons of a shirt using both hands (fleetly)	81.5%	78.7%	70.7%	68.2%	43.5%	17.82*	0.23	75.9%	55.6%	—	—	(G4 + G5) < G1
(11) Ascend stairs without using the banister (slowly)	86.3%	66.7%	58.5%	27.3%	26.1%	64.75*	0.44	63.8%	26.7%	—	—	(G4 + G5) < (G2 + G3) < G1
(17) Stand on one leg with eyes open for approximately 10–20 seconds	88.7%	74.7%	75.6%	68.2%	56.5%	19.77*	0.25	75.0%	62.2%	—	—	(G4 + G5) < G1
(32) Can carry baggage of approximately 5 kg for 10 m	91.1%	90.7%	92.7%	59.1%	60.9%	33.41*	0.32	91.4%	60.0%	—	—	(G4 + G5) < G1, (G2 + G3)
(8) Jump across a ditch of approximately 30 cm	92.3%	82.7%	85.4%	40.9%	73.9%	41.83*	0.36	83.6%	57.8%	—	—	(G4 + G5) < G1, (G2 + G3)
(20) Standing within a bus or train using the handhold	94.0%	89.3%	80.5%	68.2%	60.9%	30.50*	0.30	86.2%	64.4%	—	—	(G4 + G5) < G1
(14) Stand up from a Japanese style sitting posture using hands	95.2%	86.7%	65.9%	54.5%	56.5%	53.87*	0.41	79.3%	55.6%	—	—	(G2 + G3), (G4 + G5) < G1
(23) Put on trousers or a skirt while standing using a handhold	96.4%	92.0%	95.1%	72.7%	52.2%	55.87*	0.41	93.1%	62.2%	—	—	(G4 + G5) < G1, (G2 + G3)
(29) Roll up and put away lightweight bedding	97.0%	94.7%	95.1%	90.9%	87.0%	5.51	0.13	94.8%	88.9%	—	—	—
(1) Walk for approximately 5–10 min without taking rest	100%	96.0%	82.9%	90.9%	82.6%	30.57*	0.31	91.4%	86.7%	—	—	(G2 + G3), (G4 + G5) < G1
(10) Ascend stairs using the banister	100%	97.3%	97.6%	100%	87.0%	20.09*	0.25	97.4%	93.3%	—	—	—
(25) Do or undo the front buttons of a shirt using both hands (slowly)	100%	100%	100%	100%	100%	—	—	100%	100%	—	—	—

Note: *V*: association coefficient of Cramer, *P < 0.05/27 = 0.0019.
